# Another Sanatorium Christmas

**Published:** 1914-01-03

**Authors:** 


### Christmas Day in a Sanatorium.

As patients in sanatoria are happily not too ill, their festivities may be of an active kind. Here the festive spirit is abroad from breakfast time, when each patient finds upon his plate a useful article of clothing, gloves, socks, vests, etc. These are obtained from funds collected by the matron from her personal friends and friends of the institution. She has been collecting in good time so that she can know just what can be spent upon each gift. After breakfast many willing hands help in decorating the dining hall; thus rows of Chinese lanterns bud forth, and even the face of steady old Time himself is wreathed in gay evergreen. A kind committee member has provided turkeys for dinner, and when the plum pudding arrives well alight it has a cheer all to itself. Great excitement greets the appearance of mysterious emblems of matrimony, singleness, and riches, in the shape of thimble, rings, and threepenny bits, to say nothing of a small army of tiny china dolls, which come forth from their spicy bed in smiling nudity.

Gaiety in earnest may be said to begin at teatime; there is a mighty cracker, measuring six feet and gay of hue, occupying the centre of the table, which at the close of the meal will part asunder with a loud report and pour out a shower of good things in the form of musical instruments, toys, and jewels, all accompanied by a motto of heart-searching sentimentality or a brain-searching conundrum. There is a variety of cakes—these the gift of another committee member. The matron, doctor, and nurses will take tea with the patients, and it is the doctor and matron who will pull asunder the great cracker. After tea the company adjourns to the recreation room for games and a bran-tub. Seated by the tub is a mysterious, ancient dame, anything from ninety to a hundred. She keeps guard over the tub, stirring it silently and ensuring fair play. Someone sooner or later discovers this is nurse. The tub affords much amusement: thus, a confirmed bachelor finds himself the possessor of a pair of baby socks or box of hairpins, or a girl is provided with a complete shaving outfit. These things can be exchanged, but somehow they always keep them.

Now comes along a much-needed rest hour, and then supper time, after which another surprise yet awaits the patients. They have had no knowledge of the concert which has been preparing for some weeks past, and now songs, music, and dancing for two hours. Thus ends Christmas Day in a sanatorium.

